# Transcriptomic landscape based on annotated clinical features reveals PLPP2 involvement in lipid raft-mediated proliferation signature of early-stage lung adenocarcinoma

**DOI:** 10.1186/s13046-023-02877-w

**Published:** 2023-11-23

**Authors:** Yibei Wang, Ziwei Miao, Xiaoxue Qin, Yi Yang, Si Wu, Qi Miao, Beibei Li, Mingyu Zhang, Pengfei Wu, Yun Han, Bo Li

**Affiliations:** 1 Department of Developmental Cell Biology, Key Laboratory of Medical Cell Biology, Ministry of Education, China Medical University, Shenyang, Liaoning 110122 P. R. China; 2 Department of Thoracic Surgery, Shengjing Hospital of China Medical University, Shenyang, Liaoning 110004 P. R. China; 3 Department of Medical Oncology, The First Hospital of China Medical University, Shenyang, China; 4 Department of Laboratory Animals, China Medical University, Shenyang, China; 5 Department of Biobank, Shengjing Hospital of China Medical University, Shenyang, China; 6 Department of Radiology, The First Hospital of China Medical University, Shenyang, China; 7 Department of Radiology, Shengjing Hospital of China Medical University, Shenyang, China; 8 Department of Neurosurgery, The First Hospital of China Medical University, Shenyang, Liaoning 110001 P. R. China

**Keywords:** Early stage lung adenocarcinoma, Transcriptome sequencing, Lipid rafts, Phospholipid phosphatase 2, Cell proliferation

## Abstract

**Background:**

Image-based screening improves the detection of early-stage lung adenocarcinoma (LUAD)but also highlights the issue of high false-positive diagnoses, which puts patients at a risk of unnecessary over-treatment. Therefore, more precise discrimination criteria are required to ensure that patients with early-stage LUAD receive appropriate treatments.

**Methods:**

We integrated 158 early-stage LUAD cases from 2 independent cohorts, including 30 matched resected specimens with complete radiological and pathological information, and 128 retrospective pathological pair-samples with partial follow-up data. This integration allowed us to conduct a correlation analysis between clinical phenotype and transcriptome landscape. Immunohistochemistry was performed using tissue microarrays to examine the expression of phospholipid phosphatase 2 (PLPP2) and lipid-raft markers. Lipidomics analysis was used to determine the changes of lipid components in PLPP2-overexpressed cells. To assess the effects of PLPP2 on the malignant phenotypes of LUAD cells, we conducted mice tumor-bearing experiments and in vitro cellular experiments by knocking down PLPP2 and inhibiting lipid raft synthesis with MβCD, respectively.

**Results:**

Bioinformatics analysis indicated that the co-occurrence of lipid raft formation and rapid cell proliferation might exhibit synergistic effects in driving oncogenesis from lung preneoplasia to adenocarcinoma. The enhanced activation of the cell cycle promoted the transition from non-invasive to invasive status in early-stage LUAD, which was related to an increase in lipid rafts within LUAD cells. PLPP2 participated in lipid raft formation by altering the component contents of lipid rafts, such as esters, sphingomyelin, and sphingosine. Furthermore, elevated PLPP2 levels were identified as an independent prognostic risk factor for LUAD patients. Further results from in vivo and in vitro experiments confirmed that PLPP2 could induce excessive cell proliferation by enhancing lipid raft formation in LUAD cells.

**Conclusions:**

Our study has revealed the characteristics of gene expression profiles in early-stage LUAD patients with the different radiological and pathological subtypes, as well as deciphered transcriptomic evolution trajectory from preneoplasia to invasive LUAD. Furthermore, it suggests that PLPP2-mediated lipid raft synthesis may be a significant biological event in the initiation of early-stage LUAD, offering a potential target for more precise diagnosis and therapy in clinical settings.

**Supplementary Information:**

The online version contains supplementary material available at 10.1186/s13046-023-02877-w.

## Background

The widespread application of low-dose helical computed tomography (CT) and high-resolution, thin-section CT analysis has led to a substantial increase in the detection of pulmonary nodules, so that a higher percentage of patients will be screened out at an early-stage lung adenocarcinoma (LUAD) [1]. Large-scale clinical trial data have revealed that patients with stage-I LUAD have a favourable prognosis due to improved early-stage LUAD detection. Specifically, patients presenting with pure ground-glass opacity lesions may achieve postoperative 5-year survival rates of 95% or higher after surgical treatment [2, 3]. However, the clinical classification of early lung cancer based on imaging data also highlights the significant rate of false-positive diagnoses, thereby posing a risk to patients in terms of potential over-treatment or misdiagnosis. Moreover, certain patients might lose out on surgical opportunities due to the presence of multiple pulmonary nodules or underlying diseases. Consequently, it becomes imperative to establish more reliable discrimination criteria that enable appropriate therapeutic approaches for distinct subsets of patients with LUAD.

Recent studies have elucidated the genomic, immune, and metabolic landscape of tumor lesions in order to uncover the molecular mechanisms driving the initiation and progression of LUAD, thereby providing novel diagnostic and prognostic tools for patients [4–6]. Transcriptome sequencing has also been widely employed to identify pathogenic drivers, biomarkers, and therapeutic targets for lung cancer [7]. However, much of this research has focused on intermediate or advanced-stage cancer patients, resulting in a lack of transcriptomic information available for those with early-stage LUAD. In the presented study, to comprehensively elucidate the nature and dynamic evolution of early-stage LUAD at the molecular level, we collected the paired-samples of early-stage LUAD patients, and then performed an integrated analysis of transcriptomic features of LUAD and their association with clinical-imaging and pathological subtypes. The formation of lipid raft in the cell membrane was determined as an important biological progress for the initiation and progression of early-stage LUAD, which synergized with cell cycle to drive the development of LUAD from pre-invasiveness to minimally invasiveness and invasiveness.

Lipid rafts are small, heterogeneous, and highly dynamic lipid domains within the cell membrane that assemble specific subsets of transmembrane or glycosylphosphatidylinositol-anchored proteins and lipids such as cholesterol and sphingolipids (e.g., sphingomyelin, cerebrosides, gangliosides) [8]. Sphingomyelin and gangliosides are primarily found in lipid rafts among these categories [9, 10]. Cholesterol plays a crucial role in regulating membrane fluidity and permeability to form lipid rafts while ensuring mechanical coherence without membrane leakage [11]. Furthermore, there are two classifications of lipid rafts: flat types with flotillin as the main component; invaginated types with caveolin [12, 13]. Lipid rafts serve as a platform for recruiting signaling proteins within microdomains of the cell membrane, facilitating the initiation of signal transduction cascades and contributing to various physiological and pathological processes, including the cell cycle [14]. However, the precise role of lipid rafts in early-stage LUAD remains uncertain.

Phospholipid phosphatase family members (PLPPs) possess the ability to hydrolyze a wide range of lipid phosphates, including phosphatidate, lysophosphatidate (LPA), sphingosine 1-phosphate (S1P), ceramide 1-phosphate, and diacylglycerol pyrophosphate [15, 16]. In mammals, PLPPs are localized on various cellular membranes such as the cell membrane, endoplasmic reticulum membrane, and Golgi apparatus membrane [17]. Previous studies have indicated the presence of PLPP1 and PLPP3 in lipid rafts and caveolae within plasma membranes [18], suggesting potential implications of PLPPs in influencing the formation and function of lipid rafts. We conducted a comprehensive analysis of the lipidome and transcriptome, which revealed that phospholipid phosphatase 2 (PLPP2) played a crucial role in early-stage LUAD by promoting the formation of lipid rafts. PLPP2 has been found to be upregulated in various tumors, including colon, liver, and prostate cancer [19]. Previous studies have demonstrated that PLPP2 could regulate tumor growth by modulating cell-cycle progression; knockdown of PLPP2 in breast cancer cells resulted in reduced c-myc expression and inhibition of G1 to S phase transition [20]. However, it remains unclear whether PLPP2 is involved in LUAD onset through its impact on lipid raft formation or if it could serve as a predictor for this disease. Further investigation is needed to address these questions.

In this study, we integrated 158 early-stage LUAD cases from 2 independent cohorts, including 30 matched resect specimens with complete radiological and pathological information and 128 retrospective pathological pair-samples with part follow-up data to conduct a correlation analysis between clinical phenotype and transcriptome landscape. This integration allowed us to conduct a correlation analysis between clinical phenotype and transcriptome landscape. Our findings provided valuable insights into the trajectory of transcriptomic evolution from preneoplasia to invasive LUAD. Moreover, we had deciphered that PLPP2-mediated lipid raft synthesis might play a crucial role in driving the progression of early-stage LUAD. These results significantly contributed to our understanding of both the biological and clinical aspects of LUAD development.

## Methods

### Patients and samples

For the presented study, we included 30 patients with stage I LUAD who accepted the surgical treatment at Shengjing Hospital of China Medical University (CMU) from 2018 to 2021. Tumour tissues and paired paracancerous tissues at a distance of 5 cm from the tumour were obtained. These 30 matching specimens named as Cohort 1 were subjected to RNA sequencing. For the validation study, tumour cores and normal tissue cores (taken at least 5 cm away from the tumour) from 128 patients (named as Cohort 2) enrolled in Shengjing Hospital of CMU were included. Of these, follow-up data of 5 years were available for 80 patients with TNM tumour stages I (*n* = 42), II (*n* = 10), III (*n* = 19) and IV(*n* = 9). The 80 paired-samples were collected from these patients to construct a tissue microarray. The other paired-specimens from 48 patients with no follow-up data were also included as a validation cohort and present on the other tissue microarray. Among them, 38 pairs of samples were from patients with stage I LUAD.

### Clinical information acquisition

The pathological diagnoses, CT images, and follow-up information were obtained for each patient. The radiological characteristics of patients in Cohort 1 were reviewed by two radiologists according to the reported methods [21].

### RNA quantification and qualification

RNA concentration and purity was measured using NanoDrop 2000 (Thermo Scientific, US). RNA integrity was assessed using the RNA Nano 6000 Assay Kit of the Agilent Bioanalyzer 2100 system (Agilent Technologies, US).

### Library preparation for Transcriptome sequencing

A total amount of 1 μg RNA per sample was used as input material for the RNA sample preparations. Sequencing libraries were generated using NEBNext UltraTM RNA Library Prep Kit for Illumina (NEB, US) following manufacturer’s recommendations and index codes were added to attribute sequences to each sample. RNA sequencing was performed by the Biomarker Technologies (http://www.biomarker.com.cn/, Beijing, China).

### Clustering and sequencing

The clustering of the index-coded samples was performed on a cBot Cluster Generation System using TruSeq PE Cluster Kit v4-cBot-HS (Illumia) according to the manufacturer’s instructions. After cluster generation, the library preparations were sequenced on an Illumina platform and paired-end reads were generated.

### Quantification of gene expression levels

Gene expression levels were estimated by fragments per kilobase of transcript per million fragments (FPKM) mapped. The formula is shown as follow:

$$\mathrm{FPKM}=\frac{\mathrm{cDNAfragments}}{\mathrm{mapped}\;\mathrm{fragments}\;(\mathrm{millions})\;\times\;\mathrm{transcript}\;\mathrm{length}\;(\mathrm{kb})}$$

### Differential expression analysis

Differential expression analysis was performed using the DESeq2. The resulting P values were adjusted using the Benjamini and Hochberg’s approach for controlling the false discovery rate. Genes with an adjusted *P* value < 0.01 & fold change (FC) ≥ 2 were assigned as differentially expressed.

### Weighted gene coexpression network analysis (WGCNA)

A coexpression network of all differentially expressed genes (DEGs) was constructed by the WGCNA-R package [22] using BMK Cloud (www.biocloud.net). Briefly, the WGCNA algorithm defined the gene co-expression correlation matrix and the neighbour-joining function formed by the gene network, and then calculated the dissimilarity coefficients of different nodes and constructed the hierarchical clustering tree accordingly. The association between modules and specific phenotypes was explored to finally identify gene networks.

### Functional enrichment analysis

The annotation and visualization of the obtained biological functions and signalling pathways were performed by the Metascape database (www.metascape.org) [23]. Gene Ontology (GO), Kyoto Encyclopedia of Genes and Genomes (KEGG) pathway, hallmark gene sets, reactome gene sets, wiki-pathways, canonical pathways and PANTHER pathway were obtained according to specific DEGs.

### Lipid metabolomics detection

The detection of lipid composition was conducted by Metware Biotechnology (http://www.metware.cn/, Wuhan, China) based on the AB Sciex QTRAP 6500 LC–MS/MS platform, and the metabolomics data analysis were performed using the Metware Cloud (https://cloud.metware.cn). Significantly regulated metabolites between groups were determined by Variable Importance in Projection (VIP) ≥ 1 and absolute log2FC ≥ 1. VIP values were extracted from OPLS-DA result, which also contain score plots and permutation plots, was generated using R package MetaboAnalystR. In order to avoid overfitting, a permutation test (200 permutations) was performed. Identified metabolites were annotated using KEGG Compound database (http://www.kegg.jp/kegg/compound/), annotated metabolites were then mapped to KEGG Pathway database (http://www.kegg.jp/kegg/pathway.html). Pathways with significantly regulated metabolites mapped to were then fed into MSEA (metabolite sets enrichment analysis), their significance was determined by hypergeometric test’s P values.

### Cell lines and regents

The mouse lewis lung cancer cell line (LLC) was obtained from National Infrastructure of Cell Line Resource (NICR) and cultured in DMEM (Hyclone, US) supplemented with 10% FBS (Thermo Scientific, US). Human lung cancer cell lines (HCC827, NCI-H1975, NCI-H1299, NCI-H460, NCI-H520, NCI-H446) were obtained from American Type Culture Collection (ATCC) and cultured in RPMI 1640 (Hyclone, US) supplemented with 10% FBS. Human lung cancer cell line PC-9 was obtained from National Collection of Authenticated Cell Cultures and cultured in RPMI 1640 (Hyclone, US) supplemented with 10% FBS. Human lung cancer cell line A549 and mouse alveolar epithelial cell line (MLE-12) were obtained from ATCC and cultured in DME-F12 (Hyclone, US) supplemented with 10% FBS. Human bronchial epithelial cell line (BEAS-2B) was obtained from National Collection of Authenticated Cell Cultures and cultured in BEGM BulletKit (Lonza, Switzerland). All cells were cultured under a humidified atmosphere of 5% CO2 at 37℃. Methyl-β-cyclodextrin (MβCD) was obtained from MedChemExpress (MCE, US).

### Cell treatments

NCI-H1299 and A549 cells were pretreated with small interfering RNA (siRNA, GenePharma, China) and RNAiMAX transfection reagent (Invitrogen, US) to knock down the level of PLPP2. SiRNA-1: 5’-GGAUGUACUGCAUGGUGUUTT-3’ and siRNA-2: 5’-GCUCGGACUUCAACAACUATT-3’ were used as human PLPP2 (NM_177543.3) target sequences. Non-silencing siRNA (5’-UUCUUCGAACGUGUCACGUTT-3’) was used as the negative control. The human and mouse control / PLPP2-overexpression lentiviruses were generated by GeneChem. NCI-H1299, A549 and LLC cells were infected with homologous lentivirus particles according to the indicated procedures (GeneChem, China). NCI-H1299 and A549 cells were treated with 1 mM MβCD for 4 h to impede lipid rafts formation.

### Quantitative real-time polymerase chain reaction (Q-PCR)

TRIzol reagent (Invitrogen, US) was used to obtain total RNA according to manufacturer’s instructions. The cDNA was synthesized and amplified using a miRNA 1st Strand cDNA Synthesis Kit and miRNA Universal SYBR qPCR Master Mix kit (Vazyme, China) according to the manufacturer's protocols. The sequences of primers were listed as follows: human PLPP2 forward: 5’-GGAAACCCTGCTGATGTCACC-3’; human PLPP2 reverse: 5’-CACATACAGCGCCAAGAACAC-3’; human β-actin forward: 5’-GGGACCTGACTGACTACCTC-3’; human β-actin reverse: 5’-TCATACTCCTGCTTGCTGAT-3’. The relative levels of target genes were normalized to GAPDH by 2^ΔΔCt^ method.

### Western blot analysis

Protein extraction and Western blot were performed as we previously reported [24]. The primary antibody was rabbit PLPP2 polyclonal antibody (1:1000, OriGene TA368674, US) and rabbit β-tubulin polyclonal antibody (1: 1000, Proteintech 10068–1-AP, China). The secondary antibody was anti-rabbit IgG, HRP-linked antibody (1:10000, Cell Signalling Technology #7074, US).

### Immunofluorescence (IF)

Cells were processed for immunofluorescence staining using specific antibodies as described earlier [24]. After blocked with blocking solution for 1 h, cells were incubated with rabbit caveolin-1 monoclonal antibody (1:200, Cell Signalling Technology #3267, US) or rabbit flotillin-1 monoclonal antibody (1:50, Cell Signalling Technology #18634, US) overnight at 4℃. Secondary antibodies, Alexa Fluor 488, goat anti-rabbit (Invitrogen A32731, US) and Alexa Fluor 555, goat anti-rabbit (Invitrogen A32732, US) were used for visualization by the scanning laser confocal microscope. Nuclei were visualized using 40,6-diamidino-2-phenylindole (DAPI, Thermo Scientific, US). AOD values were calculated by imageJ software.

### Cell counting kit-8 (CCK-8) analysis and colony formation assay

For CCK-8 analysis, 2 × 103 cells were seeded into 96 well plates and stained at the indicated time-point with 10 µl CCK-8 dye (Sevenbiotech, China) for 1 h at 37˚C, followed by the absorbance measured at 450 nm. For colony formation assay, 200 cells were plated into six well plates and cultured for 10 days. Colonies were then fixed for 15 min with 4% polyformaldehyde and stained for 10 min with Crystal Violet Staining Solution (Beyotime Biotechnology, China).

### In vivo tumour growth assay

Female C57BL/6 mice (6–8 weeks old) were obtained from SPF Biotechnology (Beijing) and were subcutaneously injected with LLC/Vector or LLC/PLPP2 OE cells (2 × 10^5^ cells/mouse) suspended in 100 μl PBS. 4 days later, 100 μl PBS containing MβCD was intravenously administered to LLC/Vector and LLC/PLPP2 OE every two days for 3 weeks. The dose of MβCD used was 10 mg/kg as previously reported [25]. The number of mice in LLC/Vector, LLC/PLPP2 OE, LLC/Vector + MβCD and LLC/PLPP2 OE + MβCD was 6 respectively. Tumour volumes were monitored every 4 days. On the 24th day, mice from all groups were euthanized.

### Statistical analysis

All the data were analysed with R software (version 3.6.3, https://cran.r-project.org/), R bioconductor packages (http://www.bioconductor.org/) and GrapPad Prism9.4 software (https://www.graphpad.com/). For the comparisons of two groups, unpaired student t test was used for the unpaired variables of normal distribution. Paired student t test was used to compare the gene expression between cancer and paracancerous normal tissue. Mann–Whitney U test was used to analyse the variables of non-normal distribution. One way ANOVA were used for the comparisons of three or more groups. The Cancer Genome Atlas (TCGA) RNA-seq data and clinical information were obtained as previously described [26]. Gene Set Enrichment Analysis (GSEA) of PLPP2 was done by GSEA software (version 4.0.3, https://www.gsea-msigdb.org). The spearman correlation coefficient test was used to estimate the rank correlation among the different variables. The time-dependent receiver operating characteristic (ROC) analysis was used to evaluate the diagnostic accuracy of PLPP2 and calculate area under the curve (AUC). *P* values, two-sided, of less than 0.05 were considered statistically significant.

## Results

### Transcriptome landscape of lung preneoplasia and adenocarcinoma

In this study, we recruited a total of 158 patients who had been diagnosed with LUAD and divided them into two cohorts. The clinical data for both cohorts are presented in Table 1. Cohort 1 was designated as the testing cohort and included 30 patients with early-stage LUAD confirmed through pathological examination. Each patient provided a lung cancer tissue sample along with an adjacent non-cancerous tissue sample, resulting in a collection of 60 resected specimens that underwent high-throughput transcriptome sequencing. The obtained sequencing data were comprehensively analyzed together with the corresponding pathological and imaging information of the patients. Cohort 2, known as the validation cohort, consisted of 128 LUAD patients from whom matched pathological tissue samples and partial follow-up data were collected for laboratory testing and survival analysis purposes.

**Table 1:** Clinical information of included patients

	Cohort 1 (n%)	Cohort 2 (n%)	t/χ^2^	*P*
**Patient Number**	30	128		
**Sample Number**	60	256		
**Age**	64.13 ± 11.05	60.10 ± 9.58	2.01	0.05*
**Gender**			0.72	0.40
Female	21 (70.00)	79 (61.72)		
Male	9 (30.00)	49 (38.28)		
**Smoking status**			1.70	0.19
Non-smoker	21 (70.00)	73 (57.03)		
Smoker	9 (30.00)	55 (42.97)		
**Lesion maximum diameter (cm)**	2.04 ± 0.66	3.33 ± 1.88	-6.26	< 0.01**
**Lesion site**			10.93	0.03*
Right upper lobe	14(48.28)	31 (24.22)		
Right middle lobe	2 (6.90)	15 (11.72)		
Right lower lobe	2 (6.90)	27 (21.09)		
Left upper lobe	2 (6.90)	26 (20.31)		
Left lower lobe	9 (31.03)	29 (22.66)		
**TNM Stage**			45.86	< 0.01**
Stage 0	8 (26.67)	0 (0.00)		
Stage I	22 (73.33)	80 (62.50)		
Stage II	0 (0.00)	11 (8.59)		
Stage III	0 (0.00)	21 (16.41)		
Stage IV	0 (0.00)	16 (12.50)		

^*^P < 0.05

^**^P < 0.01

**Fig. 1:**
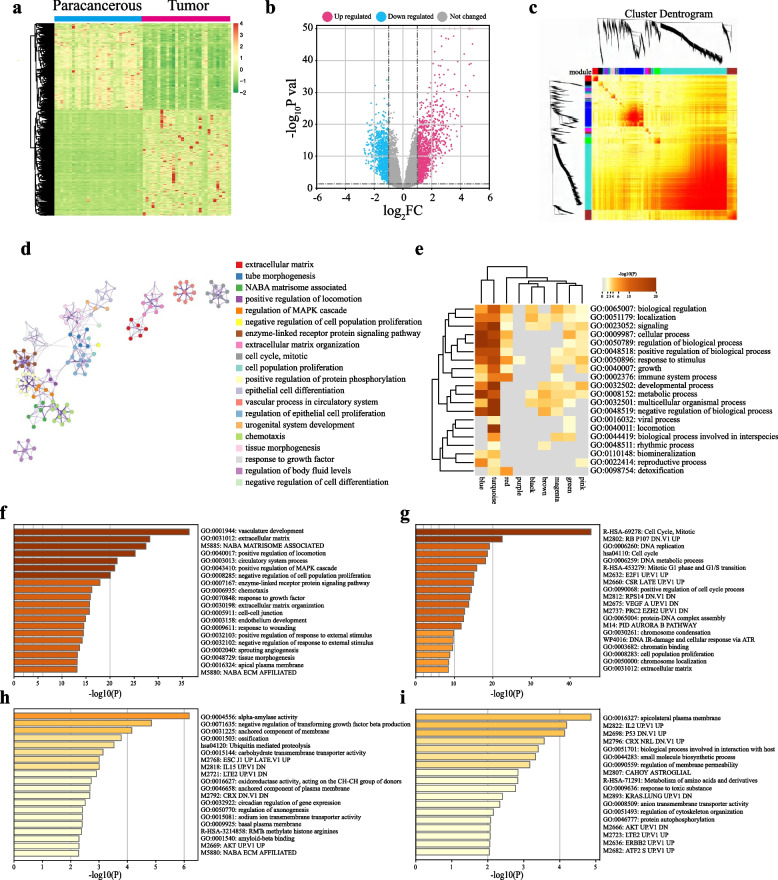
WGCNA and functional annotations for DEGs in different modules. **a** The heatmap showed the DEGs between 30 paracancerous tissues and 30 tumour tissues. **b** The volcano plot showed the DEGs between 30 paracancerous tissues and 30 tumour tissues. The red dots represented up-regulated significant DEGs and the blue dots represented sown-regulated significant DEGs. **c** WGCNA network heatmap showed cluster dendrogram and genetic similarity among different modules. **d** Functional annotations for 1827 ORDEGs. **e** Top-level gene ontology biological processes enrichment of turquoise module、blue module、brown module and green module. **f** Functional annotations for ORDEGs in turquoise module. **g** Functional annotations for ORDEGs in blue module. **h** Functional annotations for ORDEGs in brown module. **i** Functional annotations for ORDEGs in green module

The study design of our research was presented in Supplementary Figure S1, which illustrates the experimental framework. The analysis of differentially expressed genes (DEGs) was conducted on the transcriptome sequencing data obtained from patients in Cohort 1. A total of 2161 DEGs were identified, consisting of 1827 known genes and a subset of hypothetical genes (Fig. 1a and b). These genes exhibited distinct expression patterns during the transition from normal tissue to tumor tissue at early stages of LUAD onset, thus referred to as oncogenesis-related DEGs (ORDEGs). To provide a comprehensive understanding of the enriched functions represented by these ORDEGs, we employed WGCNA to establish a hierarchical clustering tree for them and utilized the dynamic-cut method to merge them into ten gene-network modules.In the co-expression network heatmap, genes within each module showed a high level of expression similarity, whereas those belonging to different modules demonstrated low similarity (Fig. 1c). Following that, correlation analysis was performed to clarify the relationships among various gene modules (Supplementary Fig. S2a and b). The results indicated a significant positive correlation between the brown and green modules (*r* = 0.73, *P* = 0.01). Additionally, there was an evident correlation observed for genes present in both brown and green modules concerning the turquoise module. These results indicated that WGCNA effectively clustered genes with high similarity into the same gene module, suggesting the establishment of a co-expression network among genes. Furthermore, there was a strong correlation between different gene modules. Subsequently, we utilized the Metascape Database to conduct functional enrichment analysis of DEGs and explored their functional interplay across various modules. We performed comprehensive functional enrichment analysis of all DEGs and identified gene subsets with similar GO terms (Fig. 1d). As expected, the significant enrichments were observed in cell cycle regulation, cell proliferation, and related pathways such as the mitogen-activated protein kinase pathway. To gain a more comprehensive understanding of the functional correlations within each gene module, we conducted functional enrichment analysis across the various gene-network modules. Our findings revealed significant enrichment for top-level GO biological processes, such as metabolic process (Fig. 1e), in the turquoise, blue, brown, and green gene modules. Subsequent correlation analysis between gene significance and module membership values revealed a positive correlation between the genes within each respective module and their corresponding functional characteristics (Supplementary Fig. S2c-f), indicating that the enriched genes in each module can effectively represent its specific functions. Furthermore, we conducted functional enrichment analysis of DEGs within their respective modules using the Metascape Database (Fig. 1f-i). It was noteworthy that specific DEGs were observed to be enriched in plasma functions, such as the apical plasma membrane within the turquoise module (Fig. 1f), anchored components of the membrane within the brown module (Fig. 1h), and apicolateral plasma membrane within the green module (Fig. 1i). Considering these findings, it could be inferred that the reorganization of cellular membrane constituents an integral part of metabolic processes and was synchronized with rapid cell proliferation to facilitate oncogenesis progression from lung preneoplasia to adenocarcinoma.

### Enhancement of cell cycle promoted the transition of early-stage LUAD from non-invasiveness to invasiveness

**Fig. 2:**
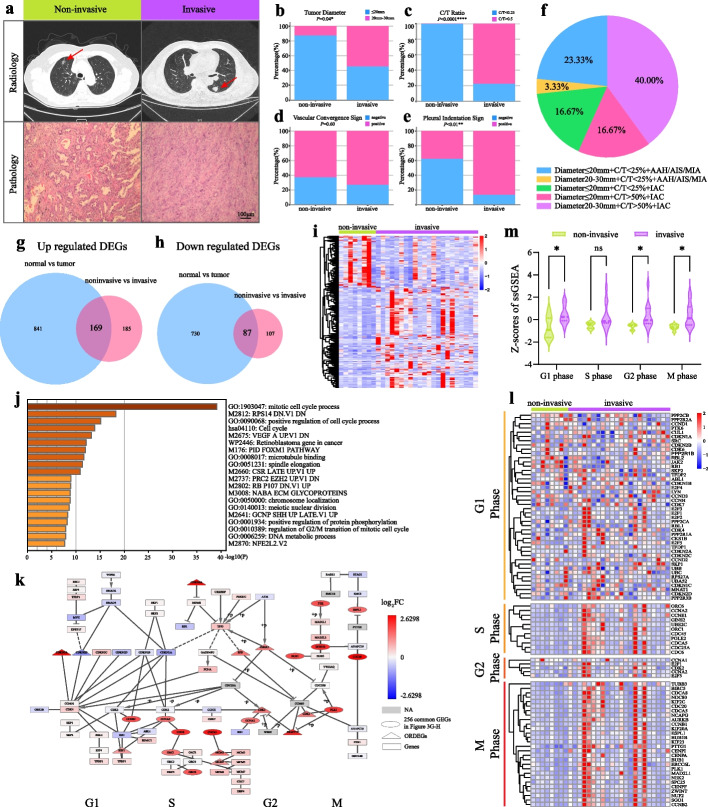
Cell-cycle activation was the primary feature of early-stage LUAD onset and progression. **a** Representative image showed the pathological and radiological features of patients in cohort 1. **b-e** Statistic analyses of tumour diameters, C/T ratios, vascular convergence sign and pleural indentation sign in patients with non-invasive and invasive nodes. *P < 0.05, **P < 0.01, ****P < 0.0001, the student’s t test. **f** Pie chart showed the percentage of patients with different pathological and radiological features. **g** Venn showed 169 common up regulated DEGs between ORDEGs and DEGs in non-invasive versus invasive group. **h** Venn showed 87 common down regulated DEGs between ORDEGs and DEGs in non-invasive versus invasive group. **i** The heatmap showed the DEGs between non-invasive and invasive tissues. **j** Functional annotations for 256 common DEGs in Fig. 3G-H. **k** Enrichment analysis of cell cycle pathway in KEGG database. **l** The heatmap showed the marker genes in G1, S, G2, M phase between non-invasive and invasive tissues. **m** Statistic analysis of ssGSEA z-scores in G1, S, G2, M phase marker genes between non-invasive and invasive tissues. *P < 0.05, ns: no significance, the student’s t test

It well known that pathological diagnosis and radiological imaging signs are important clinical index for early-stage LUAD diagnosis. To further understand the relationships between the molecular characteristics of early-stage LUAD and their different clinical phenotypes, we collected postoperative pathological diagnoses and preoperative imaging data from the 30 patients in Cohort 1 (Fig. 2a, Supplementary Table S1). Among these patients, two were diagnosed with severe atypical adenomatous hyperplasia (AAH), three with adenocarcinoma in situ (AIS), three with minimally invasive adenocarcinoma (MIA), while the remaining twenty-two patients were diagnosed with invasive adenocarcinoma (IAC). Given that AAH, AIS, and MIA lesions exhibit non-invasiveness or low invasiveness, the eight patients demonstrating these clinicopathologic characteristics were classified as the non-invasive group. The remaining 22 patients with IAC lesions were categorized as the invasive group. Subsequently, a statistical analysis was conducted to examine the correlations between imaging features and pathological diagnosis in a cohort of 30 early-stage LUAD cases. Imaging characterization of invasive nodules revealed larger tumor diameters, higher consolidation tumor (C/T) ratios, and increased indications of vascular convergence and pleural indentation when compared to their non-invasive counterparts (Fig. 2b-f). These findings demonstrated a consistent correlation between the pathological characteristics and imaging features of invasive lesions. Initially, gene expression profiles were analyzed to compare the non-invasive group with the invasive group, resulting in 548 differentially expressed genes (DEGs) that were highlighted. By intersecting these DEGs with ORDEGs, we identified 256 DEGs, out of which 169 were jointly up-regulated and 87 were jointly down-regulated. These DEGs were closely associated with the invasiveness of early-stage LUAD (Fig. 2g-i). Functional analysis of these 256 DEGs revealed a significant enrichment in mitosis and cell-cycle processes (Fig. 2j). A pathway diagram illustrating the cell cycle was constructed using the KEGG Database, revealing that a majority of differentially expressed genes (DEGs) were predominantly enriched in the S and G2/M phases of the cell cycle. These DEGs included essential components involved in replication forks during the S phase, such as ORC1, ORC6, CDC6, MCM2, MCM4, and MCM6. Furthermore, the components associated with spindle assembly checkpoint regulation during the M phase were also identified among these DEGs, including BUB1B, BUB1, PLK1, and PKMYT (Fig. 2k). Additionally, the marker genes representing distinct phases of G_1_, S, G_2_, and M were selected from the Molecular Signatures Database for both experimental groups and visualized on a heatmap (Fig. 2l). Next, single-sample gene set enrichment analysis (ssGSEA) was conducted on the gene sets according to previously described methods [27]. The results demonstrated a significant elevation in ssGSEA score within the invasive group compared to the non-invasive group (Fig. 2m). In conclusion, our findings suggested that augmented cell cycle activity might facilitate the transition from a non-invasive to an invasive state in early-stage LUAD.

### Lipid rafts-driven invasiveness of early-stage LUAD was relative to the activation of cell-cycle

**Fig. 3:**
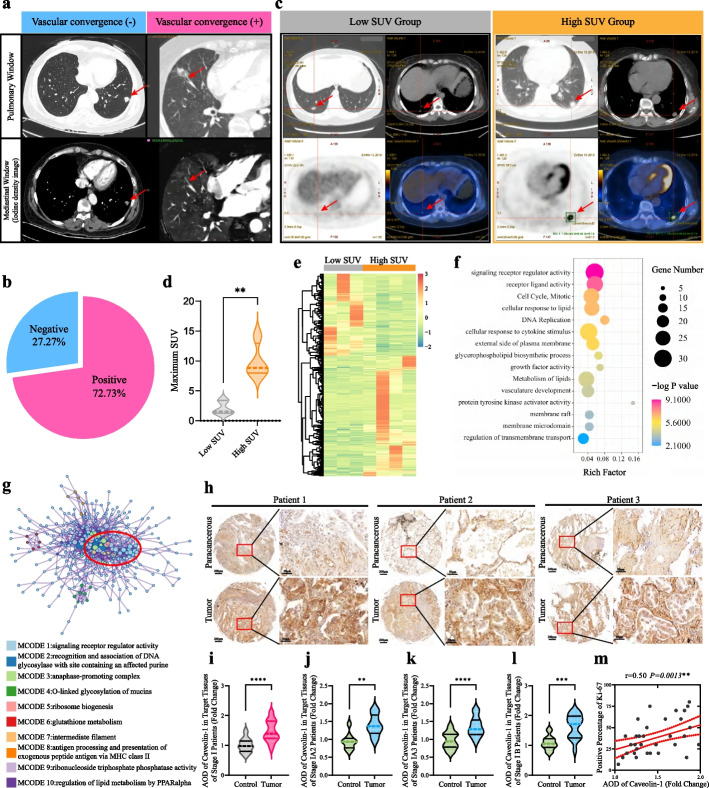
Lipid rafts drove cell-cycle activation and invasiveness in early-stage LUAD. **a** Representative image of patients with or without vascular convergence sign. **b** Pie chart showed the percentage of patients with or without vascular convergence sign in invasive group. **c** Representative PET-CT images of patients in low SUV and high SUV group. **d** Statistic analysis of patients’ maximum SUV in low SUV and high SUV group. **P < 0.01, the student’s t test. **e** The heatmap showed the DEGs between low SUV tissues and high SUV tissues. **f** Functional annotations for 475 DEGs between low SUV tissues and high SUV tissues. **g** Protein–protein interaction network among 10 densely connected components calculated by MCODE algorithm. **h** Representative images of IHC assay detecting levels of caveolin-1 expression in paracancerous and tumour tissues of LUAD patients in cohort 2. **i** Statistic analysis of caveolin-1 AOD values in paired paracancerous and tumour tissues of stage I LUAD patients in cohort 2, *n* = 38. Bars, SD; ****P < 0.0001; the student’s t test. **j** Statistic analysis of caveolin-1 AOD values in paired paracancerous and tumour tissues of stage I A2 patients in cohort 2, *n* = 9. Bars, SD; **P < 0.01, the student’s t test. **k** Statistic analysis of caveolin-1 AOD values in paired paracancerous and tumour tissues of stage I A3 patients in cohort 2, *n* = 14. Bars, SD; ****P < 0.0001, the student’s t test. **l** Statistic analysis of caveolin-1 AOD values in paired paracancerous and tumour tissues of stage I B patients in cohort 2, n = 15. Bars, SD; ***P < 0.001, the student’s t test. **m** Correlation analysis of caveolin-1 AOD fold changes and Ki-67 positive percentages in paracancerous and tumour tissues of stage I LUAD patients, *n* = 38. **P < 0.01; spearman correlation test

To gain novel insights into the underlying relationship connecting different gene signatures related to the cell cycle and alterations in the plasma membrane, a series of transcriptomic profile-based analytical methods were employed. As depicted in Fig. 1e, we observed an enrichment of metabolic processes during the initiation and progression of early-stage LUAD. Considering that metabolic processes were implicated in plasma membrane synthesis and cellular proliferation, our initial analysis focused on exploring the correlation between gene expression profiles and metabolic phenotypes stratified based on imaging characteristics in early-stage LUAD. To begin with, assessing vascular coverage through imaging served as an indicator for determining a tumor's capacity to obtain metabolites due to angiogenesis providing enough nutrients that supported its growth [28]. Out of a total of 22 IACs cases examined in this study, 6 displayed negative indications of vascular convergence while 16 demonstrated positive indications (Fig. 3a and b). Additionally, considering that standard uptake value (SUV) obtained from positron emission tomography-computed tomography (PET-CT) was also regarded as a clinical parameter used for evaluating tumor metabolism status; we gathered PET-CT data from 7 IACs cases. By analyzing both SUV values and indicators related to vascular convergence patterns among these cases; they were categorized into two distinct metabolic groups: 3 individuals fell under the low-metabolic group characterized by low SUV values along with negative indications of vascular convergence; conversely 4 individuals belonged to the high-metabolic group displaying elevated SUV values alongside positive indications of vascular convergence patterns (Fig. 3c and d). Then, a total of 475 differentially expressed genes (DEGs) were identified from the gene expression analysis (Fig. 3e). Subsequent GO enrichment analysis revealed characteristics closely associated with lipid rafts, such as membrane raft, membrane microdomain, signaling receptor regulator activity, and glycerophospholipid biosynthesis (Fig. 3f). Notably, significant alterations in mitosis were observed. These changes in the cell plasma membrane related to lipid raft formation aligned with the aforementioned findings observed during the progression from pre-invasive to minimally invasive and invasive LUAD. The available evidence suggests that inhibition of lipid raft formation may serve as a potential strategy to counteract tumor cell malignant proliferation [29]. To validate the relevance of lipid rafts and the cell cycle, we constructed a protein–protein interaction network of 475 DEGs using the molecular complex detection (MCODE) algorithm [30]. Ten densely connected network components were annotated (Fig. 3g). Among them, network component MCODE1, which was related to lipid raft-related signaling receptor regulator activity, showed significant interactions with cell cycle-related processes such as DNA glycosylase recognition binding (network component MCODE2) and anaphase-promoting complex (network component MCODE3). Interestingly, these interconnected network components align with the previously observed changes in the cell cycle (Fig. 2k). Furthermore, there was a significant positive correlation between genes related to lipid rafts and glycerophospholipid metabolism as well as genes associated with the cell cycle (Supplementary Fig. S3).

Next, in order to further validate the involvement of lipid raft formation in LUAD, we collected tissue samples from 48 patients with LUAD and their corresponding para-carcinoma tissues. Among these samples, 38 pairs were obtained from patients diagnosed with stage I LUAD. The results obtained from the tissue microarray analysis revealed significantly higher levels of caveolin-1 expression, a well-established marker for lipid rafts, in early-stage LUAD tissues compared to para-carcinoma tissues (Fig. 3h and i). Moreover, when categorizing patients with stage I lung adenocarcinoma (*n* = 38), we observed that IB stage LUAD tissues (*n* = 15) exhibited the highest level of caveolin-1 expression as compared to IA2 (*n* = 9) and IA3 stage LUAD tissues (*n* = 14) (Fig. 3j-l). Additionally, our findings demonstrated a positive correlation between Ki-67 expression, which serves as a hallmark for cell proliferation, and caveolin-1 expression in early-stage LUAD tissues (*r* = 0.5, *P* < 0.01; Fig. 3m). These observations strongly suggested that enhanced lipid raft formation might drive the progression of early-stage LUAD through activation of the cell cycle machinery.

### Integrative analysis of transcriptomics and lipidomics implicated the involvement of PLPP2 in lipid raft formation during early-stage LUAD

**Fig. 4:**
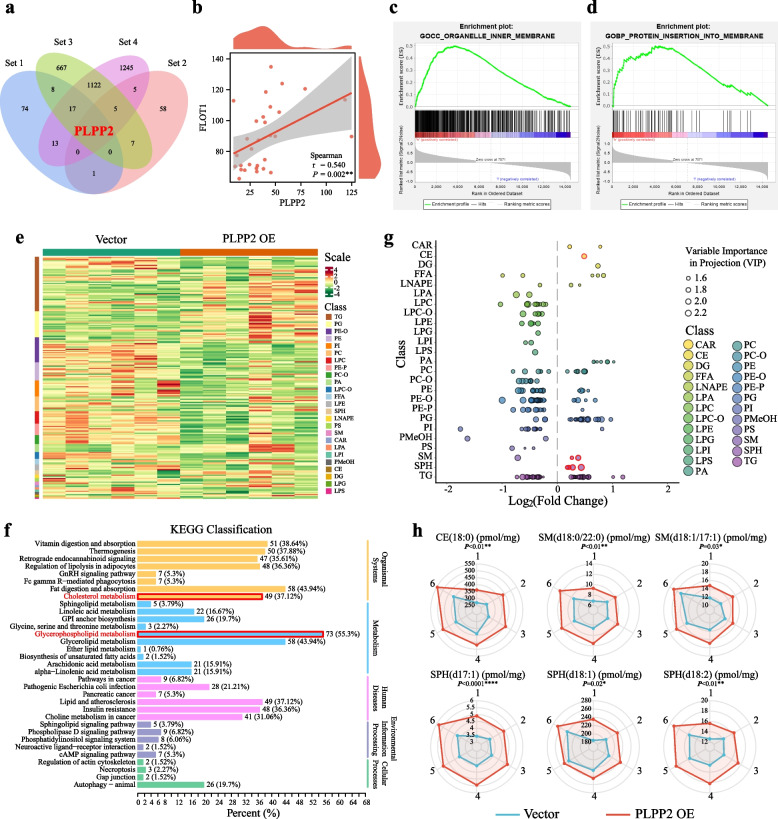
Transcriptomics and lipidomics analyses implicated PLPP2 in lipid raft formation during early-stage LUAD. **a** Venn showed common gene PLPP2 among 4 gene sets. Set 1: lipid raft gene set from molecular signatures database (MSigDB); set 2: glycerophospholipid metabolism gene set from MSigDB; set 3: ORDEGs; set 4: DEGs of paracancerous and tumour tissues of stage I LUAD patients in TCGA database. **b** Correlation analysis of PLPP2 and flotillin-1 levels in tumour tissues of patients in cohort 1, *n* = 30. **P < 0.01; spearman correlation test. **c-d** GSEA of PLPP2 levels in 30 tumour tissues of patients in cohort 1. **e** The heatmap showed differential metabolites between NCI-H1299 vector cells and PLPP2 OE cells. **f** KEGG annotation of differential metabolites between NCI-H1299 vector cells and PLPP2 OE cells. **g** Scatter plot showed the content differences of different subclasses of lipids in two groups of samples. Each point in the figure represented a type of lipid, and different colors represented different lipid subclasses. **h** Radar charts showed the levels of indicated lipids in NCI-H1299 vector cells and PLPP2 OE cells. *P < 0.05, **P < 0.01, ****P < 0.0001; the student’s t test

To investigate the key determinant underlying the formation of lipid rafts in early-stage LUAD, we initially extracted 2408 DEGs from the TCGA dataset, comprising 78 patients with stage I LUAD. By intersecting these genes with 1827 ORDEGs from Cohort 1, an additional set of 114 lipid raft-related genes, and 77 glycerophospholipid metabolism-related genes from MSigDB, we successfully identified a singular common gene known as PLPP2 (Fig. 4a). Correlation analysis conducted on samples from Cohort 1 revealed that the expression of PLPP2 exhibited a concurrent increase alongside lipid raft-associated genes such as FLOT1, CLN3, SLC2A1, and HMOX (Fig. 4b and Supplementary Fig. S4). Furthermore, gene set enrichment analysis (GSEA) demonstrated significant enrichment of PLPP2 in membrane-associated functional subsets including organelle-inner membrane and protein insertion into the membrane (Fig. 4c and d). In order to ascertain whether PLPP2 conferred to the formation of lipid raft in LUAD cells, we conducted quantitative lipidomics analysis after overexpressing PLPP2 in NCI-H1299 cells. Our analysis identified a total of 1376 lipid species, including 213 differential metabolites. Among these, 86 metabolites were found to be up-regulated while 127 were down-regulated (Fig. 4e, Supplementary Table S2). Furthermore, KEGG analysis revealed significant enrichment of signaling pathways associated with lipid raft formation, such as cholesterol metabolism and glycerophospholipid metabolism (Fig. 4f). The classification of lipid components showed that the overexpression of PLPP2 in LUAD cells could significantly increase the metabolites associated with lipid raft formation, including cholesterol esters (CEs), sphingomyelin (SM), and sphingosine (SPH) (Fig. 4g). Additionally, quantitative analysis also revealed a significant increase in the contents of CE (18:0), SM (d18:0/22:0), SM (d18:1/17:1), SPH (d17:1), SPH (d18:1), and SPH (d18:2) in NCI-H1299 cells overexpressing PLPP2 (Fig. 4h). Overall, these findings suggested that PLPP2 might participate in altering the classification and content of lipid raft components such as CE, SM, and SPH to contribute to the formation of lipid rafts in LUAD cells.

### The elevated PLPP2 was an independent prognostic risk factor for early-stage LUAD patients

**Fig. 5:**
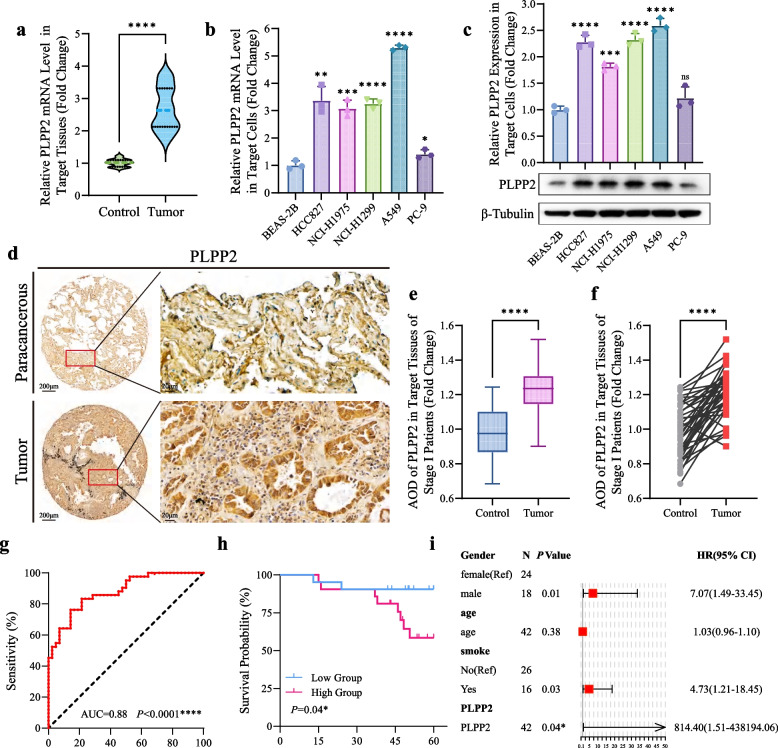
The elevated PLPP2 in LUAD served as an independent prognostic risk factor for LUAD. **a** PLPP2 mRNA levels in paired paracancerous and tumour tissues of patients in cohort 1 were tested by Q-PCR, *n* = 30. ****P < 0.0001; the student’s t test. **b** PLPP2 mRNA levels in BEAS-2B cells and LUAD cells were tested by Q-PCR, *n* = 3. Bars, SD; *P < 0.05, **P < 0.01, ***P < 0.001, ****P < 0.0001; the student’s t test. **c** The level of PLPP2 expressions in BEAS-2B cells and LUAD cells were tested by western blot, representative pictures were shown, *n* = 3. Bars, SD; ***P < 0.001, ****P < 0.0001; the student’s t test. **d** Representative images of IHC assay detecting levels of PLPP2 expression in paracancerous and tumour tissues of LUAD patients in cohort 2. **e–f** Statistic analysis of PLPP2 AOD values in paired paracancerous and tumour tissues of stage I LUAD patients in cohort 2, *n* = 42. Bars, SD; ****P < 0.0001; the student’s t test. **g** ROC analysis was used to evaluate the diagnostic accuracy of PLPP2 and calculate AUC value of stage I LUAD patients, *n* = 42. ****P < 0.0001. **h** 5-year survival probabilities of stage I LUAD patients in high and low PLPP2 groups were evaluated and showed by Kaplan–Meier curves, *n* = 42. *P < 0.05; Log-rank test. **i** Multivariate Cox hazard regression analysis of different clinical characters including age, gender and PLPP2 levels, *n* = 42

To further confirm the aforementioned findings, we employed qRT-PCR to confirm the upregulation of PLPP2 in Cohort 1 samples. As shown in Fig. 5a, the expression levels of PLPP2 mRNA were significantly elevated in LUAD tissues compared to adjacent non-cancerous tissues. Subsequently, we assessed the expression levels of PLPP2 in a panel of LUAD cells using both qRT-PCR and western blotting. Compared to BEAS-2B cells, a normal human bronchial epithelial cell line, the levels of PLPP2 in LUAD cells were significantly increased (Fig. 5b and c). To evaluate the prognostic value of the PLPP2 for LUAD patients, we used the tissues microarray to detect the levels of PLPP2 in 80 patients who was available for follow-up information in Cohort 2 (Fig. 5d). Initially, we examined the expression of PLPP2 in 42 out of the total 80 cases with stage I LUAD and discovered that it was significantly higher compared to adjacent non-cancerous tissues (Fig. 5e and f). Additionally, consistent levels of PLPP2 were observed across different TNM stages in all LUAD patients, indicating its potential as a marker for malignancy (Supplementary Fig. S5a-b). To evaluate the predictive performance, we conducted a ROC analysis and calculated the area under the ROC curves. The results revealed that PLPP2 exhibited high sensitivity and specificity in predicting LUAD, with an area under the curve (AUC) value of 0.88 (*P* < 0.0001) (Fig. 5g). Additionally, Kaplan–Meier survival analysis showed that the median survival time in the high PLPP2-group was significantly lower than that of the low PLPP2-group (hazard response [HR] = 3.68, 95% confidence interval [CI] = 1.06–12.77, *P* = 0.04; Fig. 5h). Meanwhile, the multivariate Cox regression analysis showed that elevated PLPP2 was an independent prognostic risk factor for patients with LUAD (HR = 814, 95% CI = 1.51–438,194.06, *P* = 0.04; Fig. 5i). Furthermore, consistent outcomes were observed when conducting the prognostic analysis using the complete sample of 80 patients with follow-up data in Cohort 2 (Supplementary Fig. S5). Collectively, our findings unveiled that increased PLPP2 expression independently contributed to the prognostic risk assessment of early-stage LUAD patients.

### PLPP2-induced excessive proliferation of LUAD cells were dependent on lipid rafts formation

**Fig. 6:**
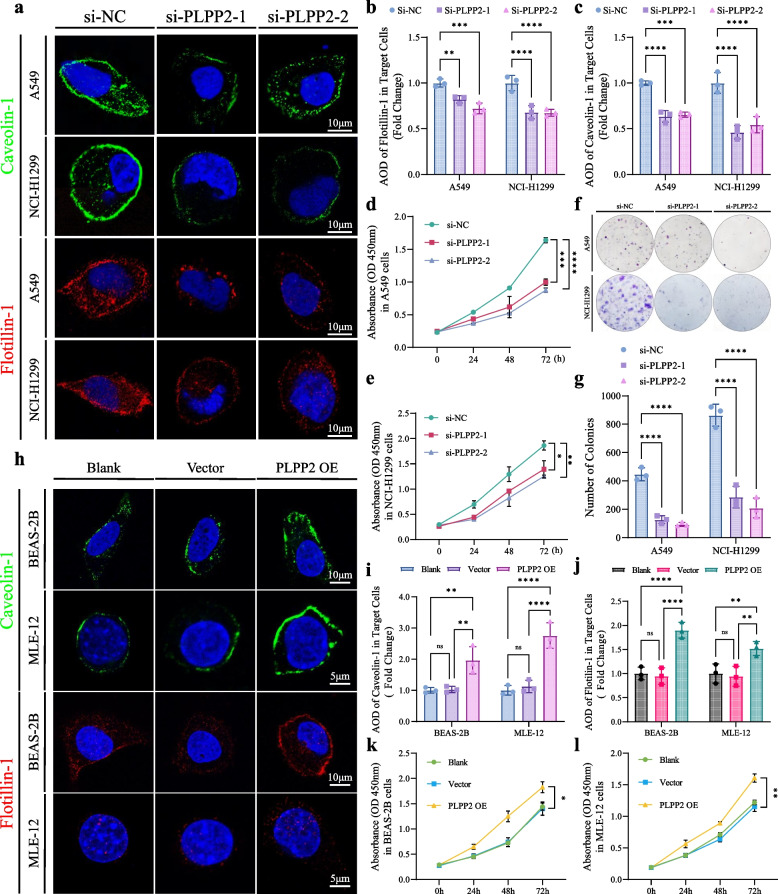
Effects of PLPP2 on the content of lipid rafts and cell proliferation. **a** A549 and NCI-H1299 cells were fixed after transfected with target siRNAs and IF was performed with antibodies recognizing flotillin-1 (red) and caveolin-1 (green). **b-c** Statistic analyses of flotillin-1 and caveolin-1 AOD values in A549 and NCI-H1299 cells after transfected with target siRNAs, *n* = 3. Bars, SD; **P < 0.01, ***P < 0.001, ****P < 0.0001; one way ANOVA. **d-e** Effects of PLPP2 knockdown on cell proliferation by CCK-8 in A549 and NCI-H1299 cells, *n* = 3. Bars, SD; *P < 0.05, ***P < 0.001, ****P < 0.0001; one way ANOVA. **f-g** Effects of PLPP2 knockdown on cell proliferation by colony formation assays in A549 and NCI-H1299 cells, *n* = 3. Bars, SD; ****P < 0.0001; one way ANOVA. **h** BEAS-2B and MLE-12 cells were fixed after transfected with target lentivirus particles and IF was performed with antibodies recognizing flotillin-1 (red) and caveolin-1 (green). **i-j** Statistic analyses of flotillin-1 and caveolin-1 AOD values in BEAS-2B and MLE-12 cells after transfected with target lentivirus particles, *n* = 3. Bars, SD; ns: no significance, **P < 0.01, ****P < 0.0001; one way ANOVA. **k-l** Effects of PLPP2 OE on cell proliferation by CCK-8 in BEAS-2B and MLE-12 cells, *n* = 3. Bars, SD; *P < 0.05, **P < 0.01; one way ANOVA

To investigate the role of PLPP2 in LUAD cells, we employed RNA-interference technology to downregulate PLPP2 expression in both A549 and NCI-H1299 cells. Immunofluorescence staining revealed a significant decrease in the localization of caveolin-1 and flotillin-1, which serve as lipid raft markers, on the cellular membrane (Fig. 6a-c). Lipid rafts played a crucial role in sensing extracellular signals that promote the malignant proliferation of tumor cells [31]. Therefore, CCK-8 assays were conducted to assess the malignant proliferation abilities of LUAD cells. Upon knockdown, both LUAD cell lines exhibited weakened proliferation capabilities (Fig. 6d-e). Similarly, the clonogenic growth assay demonstrated a significant reduction in the number of clonogenic colonies formed by LUAD cells following PLPP2 knockdown (Fig. 6f-g). Furthermore, depletion of PLPP2 also attenuated the resistance of LUAD cells to stress-induced apoptosis (Supplementary Fig. S6a-c). Additionally, transmission electron microscopy (TEM) images revealed that depletion of PLPP2 led to disruption within the inner membrane system, including compromised membrane integrity of endoplasmic reticulum and Golgi apparatus (Supplementary Fig. S6d). To further illustrate the potential of PLPP2 as a tumor marker, we employed a recombinant lentivirus to induce overexpression of PLPP2 in BEAS-2B and MLE-12 cells, which are normal lung epithelial cells. The findings revealed an elevation in caveolin-1 and flotillin-1 levels on the cell membrane (Fig. 6i-j) and enhanced cellular proliferation activity (Fig. 6k and l) subsequent to PLPP2 overexpression in normal lung cells. Furthermore, quantitative lipidomics analysis was conducted on PLPP2-overexpressed BEAS-2B cells utilizing the methodologies described in Fig. 4. Similarly, the differential metabolites were also enriched in signaling pathways associated with lipid rafts, such as cholesterol and glycerophospholipid metabolism. Furthermore, we observed a consistent pattern of alterations in the composition of lipid rafts, including SM (d18:0/22:0), SPH (d17:1), and SPH (d18:2) (Supplementary Fig. S7). Overall, our findings suggested that PLPP2 might enhance the survival of lung cancer cells by promoting proliferation and inhibiting cell death through enhancing lipid raft formation.

**Fig. 7:**
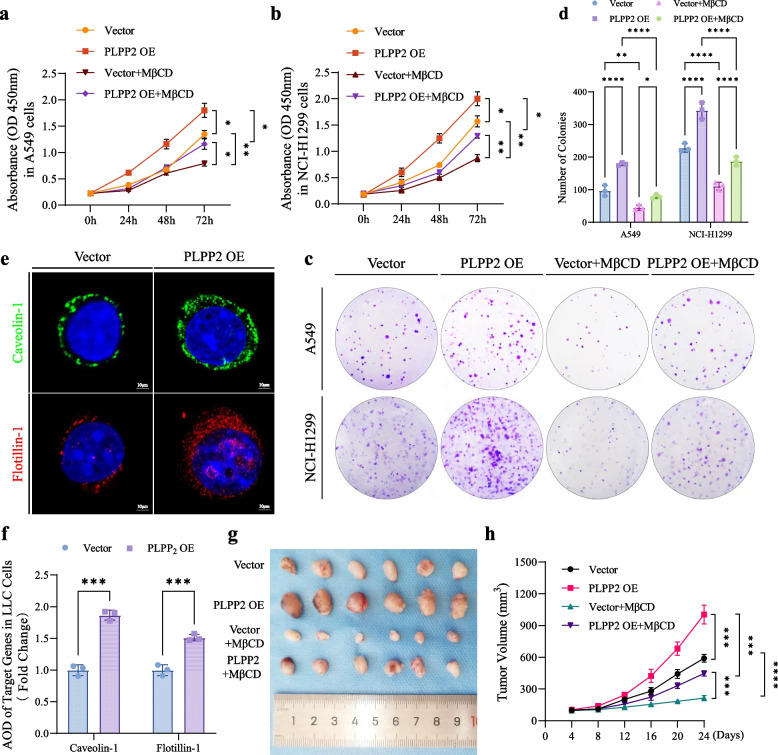
Inhibiting lipid raft synthesis in LUAD cells impeded the tumour-promoting effects of PLPP2. **a-b** Effects of PLPP2 OE on cell proliferation by CCK-8 in A549 and NCI-H1299 cells, *n* = 3. Bars, SD; *P < 0.05, **P < 0.01; two way ANOVA. **c-d** Effects of PLPP2 OE on cell proliferation by colony formation assays in A549 and NCI-H1299 cells, *n* = 3. Bars, SD; **P < 0.01, ***P < 0.001, ****P < 0.0001; two way ANOVA. **e** LLC cells were fixed after infected with target lentivirus particles and IF was performed with antibodies recognizing flotillin-1 (red) and caveolin-1 (green). **f** Statistic analyses of flotillin-1 and caveolin-1 AOD values in LLC cells after infected with target lentivirus particles, *n* = 3. Bars, SD; ***P < 0.001; the student’s t test. **g** Subcutaneous tumour images of LLC-vector group, LLC-PLPP2 OE group, LLC-vector plus MβCD treatment group and LLC-PLPP2 OE plus MβCD treatment group in C57BL/6 mice. **h** Tumour growth curves derived from target groups were shown, *n* = 6. Bars, SD; ***P < 0.001, ****P < 0.0001; two way ANOVA

To further investigate the role of PLPP2 in promoting the malignant phenotype of LUAD cells through a lipid raft-dependent mechanism, we utilized a recombinant lentivirus to overexpress PLPP2 in human A549 and NCI-H1299 cells, as well as mouse LLC cells. Subsequently, we employed MβCD, a lipid raft-synthesis inhibitor, in subsequent experiments to reduce the abundance of lipid rafts on the membrane of LUAD cells [32]. We then assessed the impact of PLPP2 on the malignant phenotype of LUAD cells and observed that MβCD effectively inhibited excessive proliferation induced by PLPP2 overexpression (Fig. 7a and b). Similarly, application of a lipid raft inhibitor completely abrogated PLPP2-induced clonal overgrowth in LUAD cells (Fig. 7c and d). Immunofluorescence analysis revealed a significant increase in lipid raft contents in mouse LLC cells overexpressing PLPP2 (Fig. 7e and f). Next, the impact of PLPP2 on tumorigenesis was further examined in mice harboring subcutaneous tumors. As illustrated in Fig. 7g and h, overexpression of PLPP2 in LLC cells significantly enhanced tumor formation in mice. The group with PLPP2 overexpression exhibited notably larger tumor volume and largest tumor area compared to the vector-control group. However, treatment with MβCD effectively inhibited tumor growth in vivo, leading to significant reductions in both tumor volume and transverse largest area (Supplementary Fig. S8a and b). Immunohistochemistry (IHC) analysis was employed to evaluate lipid raft contents within the tumor tissue. Following PLPP2 overexpression, a marked increase was observed in caveolin-1 and flotillin-1 expression levels among mice bearing tumors; nevertheless, these effects were completely abrogated by the inhibitor of lipid raft (Supplementary Fig. S8c-e). Cell proliferation in tumor tissues was assessed by analyzing the percentage of Ki-67 and proliferating cell nuclear antigen (PCNA) positivity. MβCD significantly mitigated the overexpression of PLPP2-induced excessive proliferation, characterized by high-positive staining of Ki-67 and PCNA in tumor tissues (Supplementary Fig. S8f-h). Furthermore, there was a positive correlation between the levels of lipid rafts in tumor tissues and cell proliferation (Supplementary Figure S8i). These results from inhibition experiments further validated that PLPP2 might induce excessive proliferation of LUAD cells through its potential promotion of lipid raft formation.

## Discussion

Recently, many research groups had employed next-generation sequencing (NGS) to analyse the genomic profiles of early-stage LUAD samples. Such efforts made for the understanding of early lung cancer initiation and progression had largely focused on profiling of cancer cells with genetic aberrations [4]. The joint analysis of an whole-exome sequencing and radiomics data revealed that the somatic-mutation rate in patients with early-stage LUAD was 1.12 mutations/Mb and the branched evolution and significant genomic heterogeneity were exhibited in subsolid nodules (SSNs) [33]. In the other study, a comparison of the whole-exome and transcriptome profiles of AIS and MIA tissues revealed that several genetic and immune features have been identified to drive genomic instability and clonal evolution within LUAD cancers [34]. Here, we integrated 158 early-stage LUAD cases from 2 independent cohorts, 128 pair-tissue samples and 30 matched samples with complete radiological and pathological information to conduct a correlation analysis between clinical phenotype and transcriptome landscape. We deciphered the transcription profiles of evolution trajectories from pre-invasive to minimally invasive and invasive LUAD, providing complementary insights beyond the current genomic understanding. In the correlation analysis, molecular features were found to offer important information beyond clinical features in LUAD patient stratification. Intriguingly, we found the malignant progression of early-stage LUAD from low invasiveness to high invasiveness was accompanied by the enhancement of lipid rafts-related functions. Moreover, the co-occurrence of plasma membrane changes and rapid cell proliferation might exhibit a synergistic effect to drive the development of LUAD. Our findings might provide a comprehensive understanding of the nature evolution pathways of LUAD, which contribute to precise diagnosis and treatment in patients with LUAD.

The cell membrane plays a pivotal role in the regulation of cellular functions, particularly in signal transduction [35]. Lipid rafts as selective and dynamic platforms for recruiting or excluding signaling molecules to/from the cell membrane, based on changes in size and composition. The presence of lipid rafts not only effectively safeguards signal molecules from degradation but also ensures efficient delivery of extracellular signals into the cell [14, 36, 37]. Numerous studies had demonstrated that lipid rafts govern cancer cell survival and proliferation by modulating signal transduction processes such as the insulin-like growth factor system and phosphatidylinositol 3-kinase-AKT pathway [38]. Similarly, based on the clues obtained from bio-informatics analysis, we have discovered that the rearrangement of cellular membrane components and cell proliferation were prevalent occurrences in early-stage LUAD. Moreover, this rearrangement was characterized by the formation of lipid rafts within the cell membrane. Subsequent experimental data further supported a significant correlation between elevated lipid content in LUAD cells' rafts and the progression of early-stage LUAD. Previous research had demonstrated that lipid rafts indirectly regulate the cell cycle by modifying the distribution of signaling molecules within membrane microdomains. In light of our study, it became evident that there existed a functional connection between lipid rafts and the cell cycle during early-stage LUAD, shedding light on how these lipid rafts might directly impact regulators involved in controlling cellular division through alternative pathways such as vesicle endocytosis and trafficking. Our study collectively demonstrated that the presence of lipid rafts in early-stage LUAD was an essential prerequisite for initiating downstream pro-cancer signaling pathways. This occurrence played a pivotal role as an initial trigger for activating cell-cycle progression and promoting invasiveness.

PLPPs are a family of integral membrane glycoproteins that play crucial roles in diverse physiological and pathological processes, encompassing angiogenesis, cell-cycle regulation, cardiovascular diseases, and cancer progression. These proteins function as catalysts for the dephosphorylation of lysophosphatidic acid (LPA) and sphingosine 1-phosphate (S1P), thereby terminating their signal transduction cascades. Emerging evidence had shed light on the distinct effects exerted by different members of the PLPPs family on various tumor types. Specifically, the reduced expression levels of PLPP1 and PLPP3 had been observed in colon cancer and breast cancer. Overexpression of these two isoforms promoted the hydrolysis of extracellular LPA, resulting in impaired colony formation in ovarian cancer cells [39, 40]. These studies indicated that PLPP1 and PLPP3 played tumor-suppressive roles. Conversely, elevated levels of PLPP2 have been observed in many cancers, including liver and prostate [41], breast [42] and renal cell cancer [43], which was opposite to the expression pattern of PLPP1 and PLPP3. Therefore, PLPP2 was also considered an indicator of poor prognosis. In vitro experiments had also shown that knockdown of PLPP2 weakened the growth of anchor-dependent cancer cell lines and reduced cell proliferation by delaying S-phase entry, unlike what was seen with PLPP1 and PLPP3 [41, 44]. In the presented study, we discovered that PLPP2 played a significant role in the formation of lipid rafts, leading to enhanced survival of LUAD cells. Firstly, through an analysis of transcriptomic data from matched samples of early-stage LUAD patients combined with TCGA data, we identified PLPP2 as a potential crucial factor influencing lipid raft formation in early-stage LUAD. Secondly, our lipidomics analysis further revealed substantial enrichment of lipid raft components such as CEs, SM, and SP when overexpressing PLPP2 in both LUAD cells and normal lung cells. Additionally, the examination of TCGA database also demonstrated that elevated levels of PLPP2 were an independent prognostic risk factor for patients with LUAD. Therefore, we propose that the involvement of PLPP2 in lipid raft formation contributes significantly to the development of LUAD. Subsequent in vivo and in vitro experiments provided further evidence supporting the role of PLPP2 in promoting cell proliferation and tumor growth through its influence on lipid raft formation. However, the precise mechanisms by which PLPP2 regulates lipid raft formation remain elusive. We postulated that PLPP2 regulated the assembly of lipid rafts through two distinct mechanisms: firstly, by virtue of its ecto-activity, plasma membrane-bound PLPP2 induced the dephosphorylation of S1P, leading to dynamic alterations in membrane rafts and enhanced co-localization with p-caveolin-1 [45]. Secondly, intracellular PLPP2 localized on organelles such as the ER and Golgi apparatus, exerting its influence on diverse intracellular pools of substrates to directly or indirectly modulate intracellular signaling transduction and contributed to lipid raft formation. Consequently, it is imperative for us to further investigate the precise underlying mechanism in future studies.

As anticipated, our findings have also revealed that the activation of the cell cycle is an initial event contributing to the transition of LUAD cells from dormancy to an active state. Nevertheless, there are certain unique aspects when compared to the previous study's results [46]. The majority of DEGs obtained from our transcriptomic data primarily showed enrichment during the S and G2/M phases of the cell cycle. These include key components like ORC1, ORC6, CDC6, MCM2, MCM4 and MCM6 which are involved in replication forks; along with BUB1B, BUB1, PLK1, and PKMYT associated with spindle assembly checkpoints. This emphasizes that defects occurring during mitosis such as DNA replication issues during S phase and abnormalities at spindle assembly checkpoints during G2/M phase may significantly contribute towards driving early-stage LUAD development.

It should be pointed out that our study had two primary limitations: (1) Due to the limited sample collection, our focus was solely on examining genetic profile alterations in early-stage LUAD. Therefore, future investigations will necessitate multi-center clinical studies with much more samples. (2) Further research will be required for exploring the underlying molecular mechanism of how PLPP2 control lipid rafts synthesis in LUAD cells.

## Conclusions

In summary, our study elucidated the characteristics of gene expression profiles in early-stage LUAD patients with different radiological and pathological subtypes and deciphered the transcriptomic evolution trajectory from preneoplasia to invasive LUAD. Our findings provide new implications regarding the application value of molecular-radiology-pathology-omics fusion diagnosis in tumor occurrence and progression. Additionally, for the first time, our data suggest that PLPP2-mediated lipid raft synthesis may be a significant biological event in early-stage LUAD, offering a potential target for more precise diagnosis and therapy in clinical settings.

### Supplementary Information

**Additional file 1.**

## Data Availability

The data used and/or analysed during the current study are available from the corresponding author on reasonable request. Transcriptome sequencing raw and processed data of 60 samples are available in NCBI GEO (GSE233774).

## Abbreviations

AAH: Atypical adenomatous hyperplasia
AIS: Adenocarcinoma in situ
AUC: Area under the curve
C/T: Consolidation tumour
CCK-8: Cell counting kit-8
CEs: Cholesterol esters
CT: Computed tomography
DEGs: Differentially expressed genes
FC: Fold change
FPKM: Fragments per kilobase of transcript per million fragments
GO: Gene Ontology
GSEA: Gene set enrichment analysis
IAC: Invasive adenocarcinoma
IF: Immunofluorescence
IHC: Immunohistochemistry
KEGG: Kyoto Encyclopedia of Genes and Genomes
LPA: Lysophosphatidate
LUAD: Lung adenocarcinoma
MCODE: Molecular-complex detection
MIA: Minimally invasive adenocarcinoma
MβCD: Methyl-β-cyclodextrin
NGS: Next-generation sequencing
ORDEGs: Oncogenesis-related DEGs
PCNA: Proliferating cell nuclear antigen
PLPP2: Phospholipid phosphatase 2
Q-PCR: Quantitative real-time polymerase chain reaction
ROC: Receiver operating characteristic
S1P: Sphingosine 1-phosphate
siRNA: Small interfering RNA
SM: Sphingomyelin
SPH: Sphingosine
SSNs: Subsolid nodules
SUV: Standard uptake value
TEM: Transmission electron microscopy
TNM: Tumour-node-metastasis
VIP: Variable Importance in Projection
WGCNA: Weighted gene coexpression network analysis
