# Supplementary material for: Transcriptomic landscape based on annotated clinical features reveals PLPP2 involvement in lipid raft-mediated proliferation signature of early-stage lung adenocarcinoma
Source: J Exp Clin Cancer Res. 2023 Nov 23;42:315. doi: 10.1186/s13046-023-02877-w (PMC10666437; doi:10.1186/s13046-023-02877-w)

Supplementary Figure S1

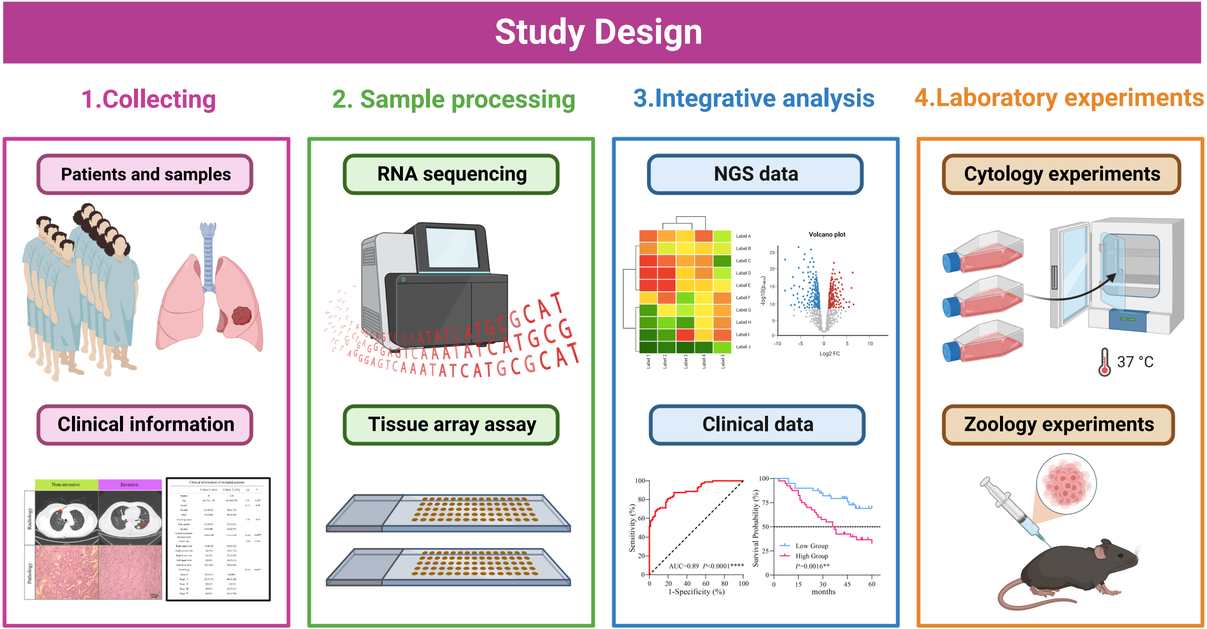

Supplement: Supplementary file 1 — Additional file 1. [file 13046_2023_2877_MOESM1_ESM.zip › Figure S1.pdf]
