# Supplementary material for: Transcriptomic landscape based on annotated clinical features reveals PLPP2 involvement in lipid raft-mediated proliferation signature of early-stage lung adenocarcinoma
Source: J Exp Clin Cancer Res. 2023 Nov 23;42:315. doi: 10.1186/s13046-023-02877-w (PMC10666437; doi:10.1186/s13046-023-02877-w)
Supplement: Supplementary file 1 — Additional file 1. [file 13046_2023_2877_MOESM1_ESM.zip › Supplementary Information.docx]

**Supplementary Information**

**1. Supplementary Figure Legends**

***Supplementary Figure S1: Schematic of the study design, created with BioRender.com (agreement number: VI25GXGEHT).***

***Supplementary Figure S2: Correlation analyses of WGCNA modules.***

**a** Eigengene adjacency heatmap showed the correlation among WGCNA modules.

**b** Functional correlation among WGCNA modules.

**c-f** Correlation analyses of eigengene connectivity (KME) values and gene significance in turquoise module, blue module, brown module and green module.

***Supplementary Figure S3: Correlation analysis of cell cycle genes and lipid raft genes or glycerophospholipid metabolism genes.***

**a** Expression correlations of lipid raft genes and cell cycle genes in 7 invasive tumour tissues, **P* < 0.05, spearman correlation test.

**b** Expression correlations of glycerophospholipid metabolism genes and cell cycle genes in 7 invasive tumour tissues, **P* < 0.05, spearman correlation test.

***Supplementary Figure S4: Correlation analysis of PLPP2 and lipid raft genes.***

**a-c** Expression correlations of PLPP2 and CLN3, SLC2A1, HMOX1 levels in tumour tissues of patients in cohort 1, n=30. **P* < 0.05, ***P* < 0.01; spearman correlation test.

***Supplementary Figure S5: ROC and survival analysis of patients in cohort 2.***

**a** Statistic analysis of PLPP2 AOD values in paired paracancerous and tumour tissues of LUAD patients in cohort 2, n=80. Bars, SD; *****P* < 0.0001; the student’s t test.

**b** Statistic analysis of PLPP2 AOD values in tumour tissues of TNM stage Ⅰ-Ⅳ, n=80. Bars, SD; **P* < 0.05; the student’s t test.

**c** ROC analysis was used to evaluate the diagnostic accuracy of PLPP2 and calculate AUC value, n=80. *****P* < 0.0001.

**b** 5-year survival probabilities of patients in high and low PLPP2 groups were evaluated and showed by Kaplan-Meier curves, n=80. **P* < 0.05; Log-rank test.

**c** Multivariate Cox hazard regression analysis of different clinical characters including age, gender, TNM stage, and PLPP2 levels, n=80.

***Supplementary Figure S6: Effects of PLPP2 knockdown on apoptosis.***

**a** Representative image of flow cytometry analysis.

**b-c** Statistic analyses of apoptosis assay in A549 and NCI-H1299 cells after transfected with target siRNAs, n=3. Bars, SD; **P* < 0.05, ***P* < 0.01, ****P* < 0.001, ns: no significance; one way ANOVA.

**d** Effects of PLPP2 knockdown on cell endoplasmic reticulum indicated by TEM in NCI-H1299 cells.

***Supplementary Figure S7: Lipidomics analysis of BEAS-2B cells.***

**a** The heatmap showed differential metabolites between BEAS-2B vector cells and PLPP2 OE cells.

**b** KEGG annotation of differential metabolites between BEAS-2B vector cells and PLPP2 OE cells.

**c** Bar charts showed the levels of indicated lipids in BEAS-2B vector cells and PLPP2 OE cells. **P* < 0.05, *****P* < 0.0001; the student’s t test.

***Supplementary Figure S8: Expression analysis of lipid raft genes and cell proliferation genes in tumours of C57BL/6 mice.***

**a** HE staining images of tumours in LLC-vector group, LLC-PLPP2 OE group and LLC-PLPP2 OE plus MβCD treatment group.

**b** Statistic analysis results showed maximum tumour areas in target groups, n=6. Bars, SD; ****P* < 0.001, *****P* < 0.0001; one way ANOVA.

**c** Representative images of IHC assay detecting levels of caveolin-1 and flotillin-1 expression in tumours of target groups.

**d-e** Statistic analysis of caveolin-1 and flotillin-1 AOD values in tumours of target groups, for each sample 3 different visual fields were counted, n=18. Bars, SD; *****P* < 0.0001; one way ANOVA.

**f** Representative images of HE staining and IHC assay detecting levels of Ki-67 and PCNA expression in tumours of target groups.

**g-h** Statistic analysis of Ki-67 and PCNA AOD values in tumours of target groups, for each sample 3 different visual fields were counted, n=18. Bars, SD; *****P* < 0.0001; one way ANOVA.

**i** Expression correlation of lipid raft genes and cell proliferation genes in tumours of C57BL/6 mice. For each sample 3 different visual fields were counted, n=18. **P* < 0.05, ***P* < 0.01, ****P* < 0.001; spearman correlation test.

**2. Supplementary Table Legends**

***Supplementary Table S1: Pathological and radiological information of patients in Cohort 1.***

***Supplementary Table S2: Lipids contents in NCI-H1299 and BEAS-2B cells infected with vector or PLPP2 OE lentivirus particles.***

**3. Supplementary Methods**

***Immunohistochemistry (IHC)***

IHC was performed following the protocol provided by Rabbit ABC detection kit (ZSGB-BIO, China). The sections were incubated overnight at 4℃ with primary antibodies: rabbit PLPP2 polyclonal antibody (1:100, OriGene TA368674, US), rabbit caveolin-1 monoclonal antibody (1:400, Cell Signaling Technology #3267, US), rabbit flotillin-1 monoclonal antibody (1:100, Cell Signaling Technology #18634, US), rabbit Ki67 polyclonal antibody (1:100, Absin abs130135, China), rabbit PCNA polyclonal antibody (1:1000, Proteintech 10205-2-AP, China). Average optical density (AOD) values were calculated by image-pro plus 6 software.

***Hematoxylin and eosin (H&E) stain***

Tumour samples were obtained and cut into 5 μm thick sections after 4% polyformaldehyde fixation, alcohol dehydration and paraffin immersing. Tumour sections were subjected to hematoxylin and eosin (H&E) stain (ZSGB-BIO, China) according to indicated protocols.

***Apoptosis detection assay***

According to the previous study[1], NCI-H1299 and A549 cells transfected with siRNAs were subjected to apoptosis assay by annexin V-FITC apoptosis detection kit (Beyotime Biotechnology, China) in the presence of H_2_O_2_ (100 mM) for 24 h.

***Transmission electron microscopy (TEM)***

TEM samples were prepared as previously described[2]. Briefly, NCI-H1299 cells were transfected with siRNAs, then pre-fixed in 4℃ overnight using 2.5% glutaraldehyde. The cells were washed in 0.1 M sodium cacodylate buffer (SCB) three times and postfixed in 1% osmium tetroxide in 0.1 M SCB at room temperature for 1 h, then were dehydrated through a graded series of ethanol and 100% acetone. The dehydrated cells were then infiltrated with acetone-Epon 812 resin mixtures and 100% Epon 812 resin. Ultra-thin serial sections were collected on copper formvarcoated slot grids, stained with 2% uranyl acetate and lead citrate, and visualized under electron microscope (H7650, Hitachi).

***References***

1. Xu ZH, Miao ZW, Jiang QZ, Gan DX, Wei XG, Xue XZ, et al. Brain microvascular endothelial cell exosome-mediated S100A16 up-regulation confers small-cell lung cancer cell survival in brain. Faseb j. 2019;33(2):1742-57.

2. Zhao WD, Liu DX, Wei JY, Miao ZW, Zhang K, Su ZK, et al. Caspr1 is a host receptor for meningitis-causing Escherichia coli. Nat Commun. 2018;9(1):2296.
