# Supplementary figures and images for: Transcriptomic landscape based on annotated clinical features reveals PLPP2 involvement in lipid raft-mediated proliferation signature of early-stage lung adenocarcinoma
Source: J Exp Clin Cancer Res. 2023 Nov 23;42:315. doi: 10.1186/s13046-023-02877-w (PMC10666437; doi:10.1186/s13046-023-02877-w)

# Supplementary Figure S2

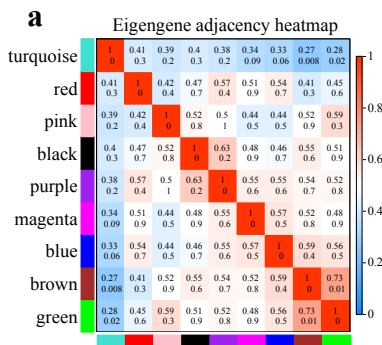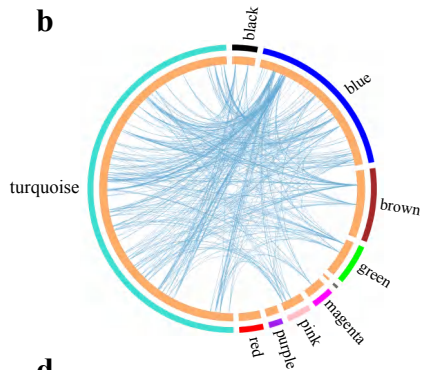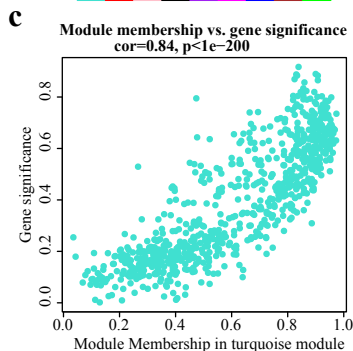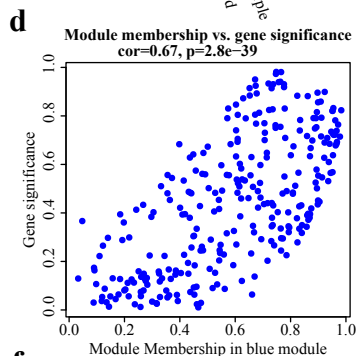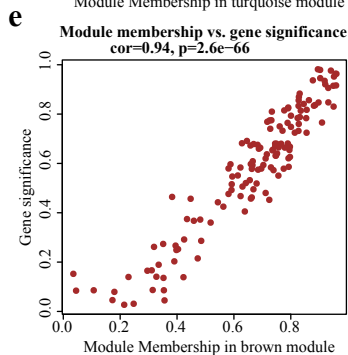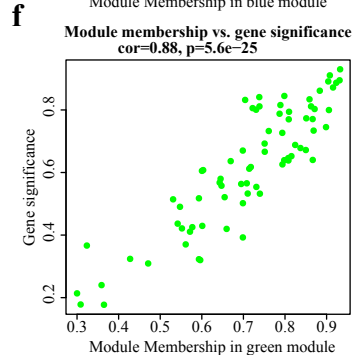

Supplement: Supplementary file 1 — Additional file 1. [file 13046_2023_2877_MOESM1_ESM.zip › Figure S2.pdf]

## Supplementary Figure S4

**a**

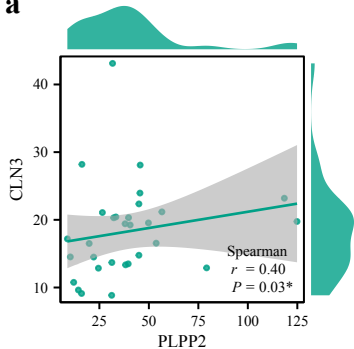

**b**

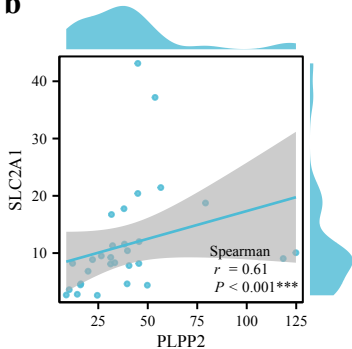

**c**

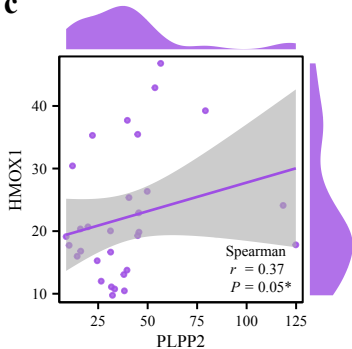

Supplement: Supplementary file 1 — Additional file 1. [file 13046_2023_2877_MOESM1_ESM.zip › Figure S4.pdf]

# Supplementary Figure S5

**a**

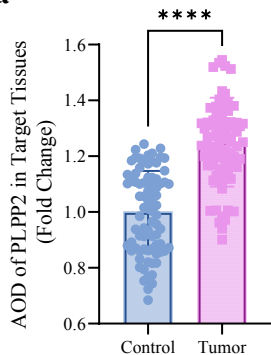

**b**

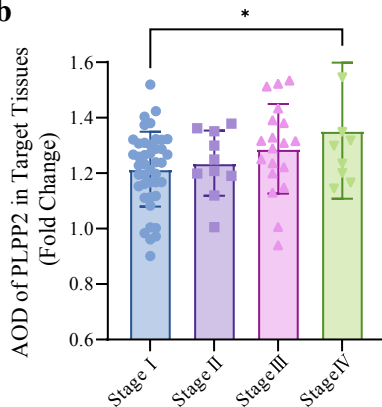

**c**

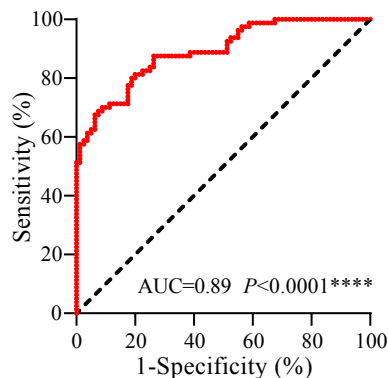

**d**

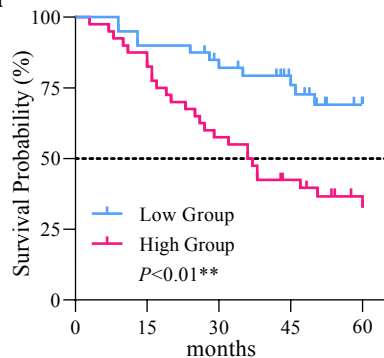

**e**

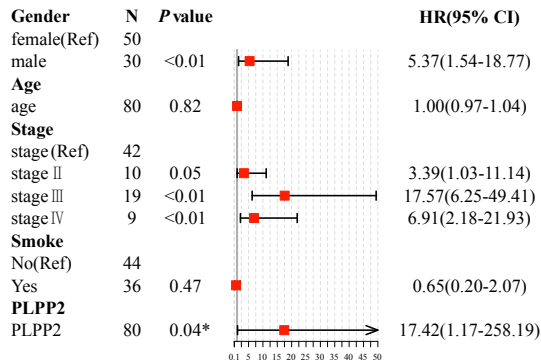

Supplement: Supplementary file 1 — Additional file 1. [file 13046_2023_2877_MOESM1_ESM.zip › Figure S5.pdf]

# Supplementary Figure S6

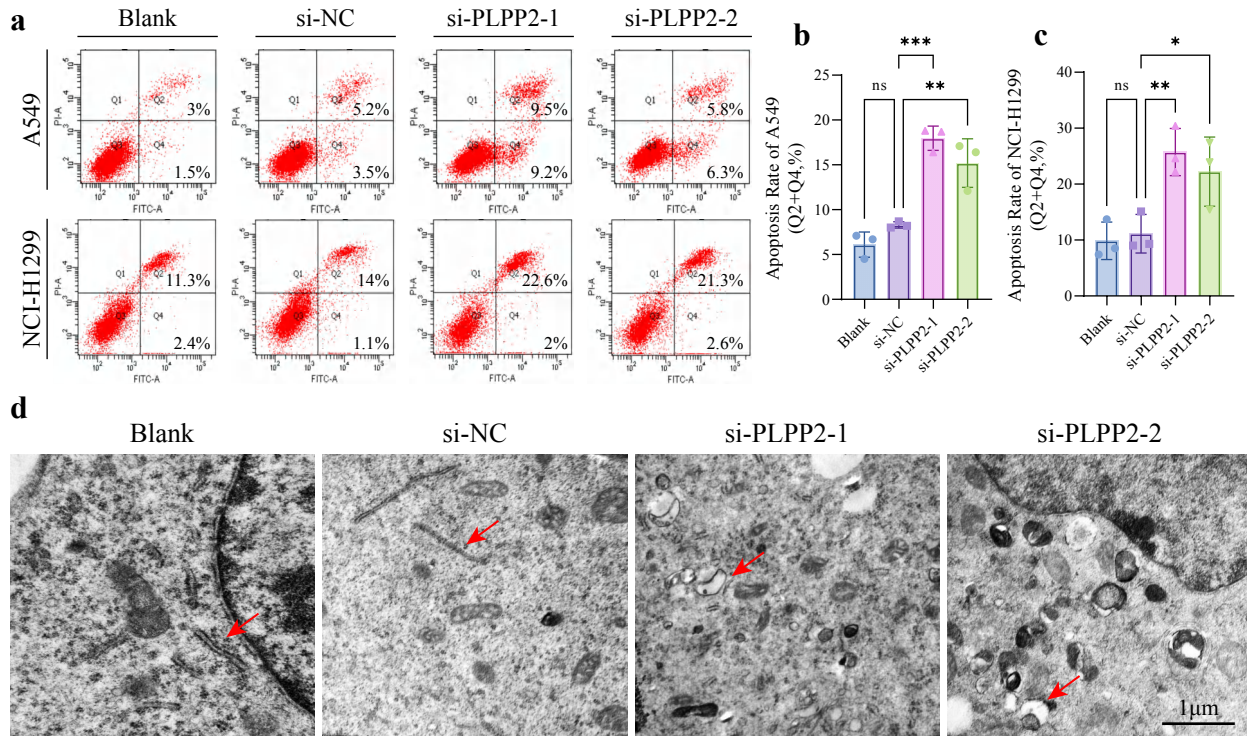

Supplement: Supplementary file 1 — Additional file 1. [file 13046_2023_2877_MOESM1_ESM.zip › Figure S6.pdf]

# Supplementary Figure S7

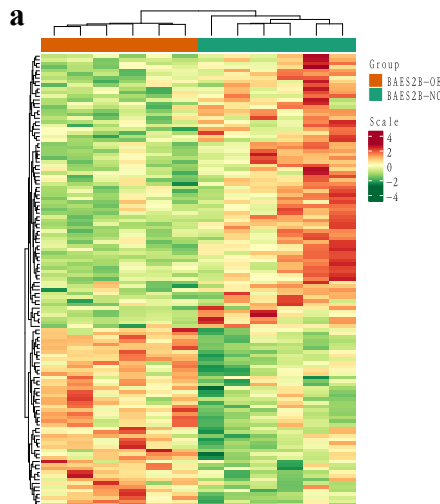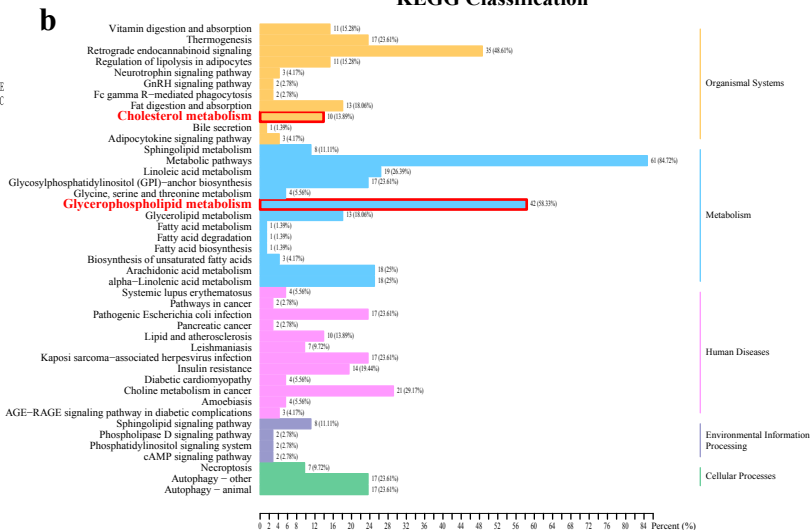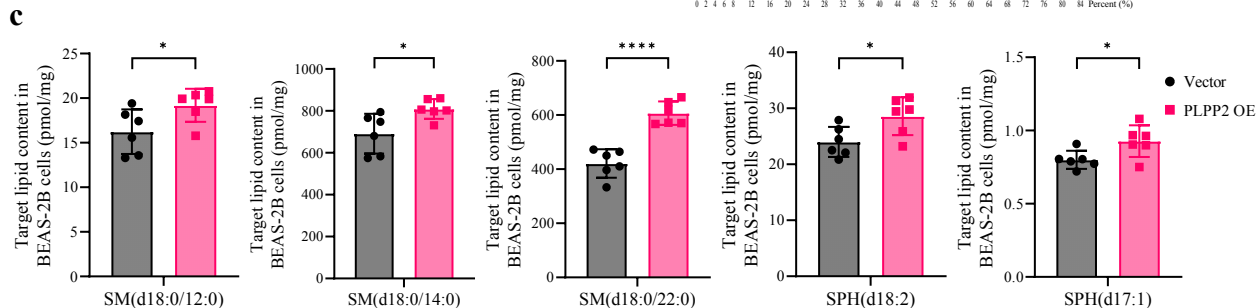

Supplement: Supplementary file 1 — Additional file 1. [file 13046_2023_2877_MOESM1_ESM.zip › Figure S7.pdf]

# Supplementary Figure S8

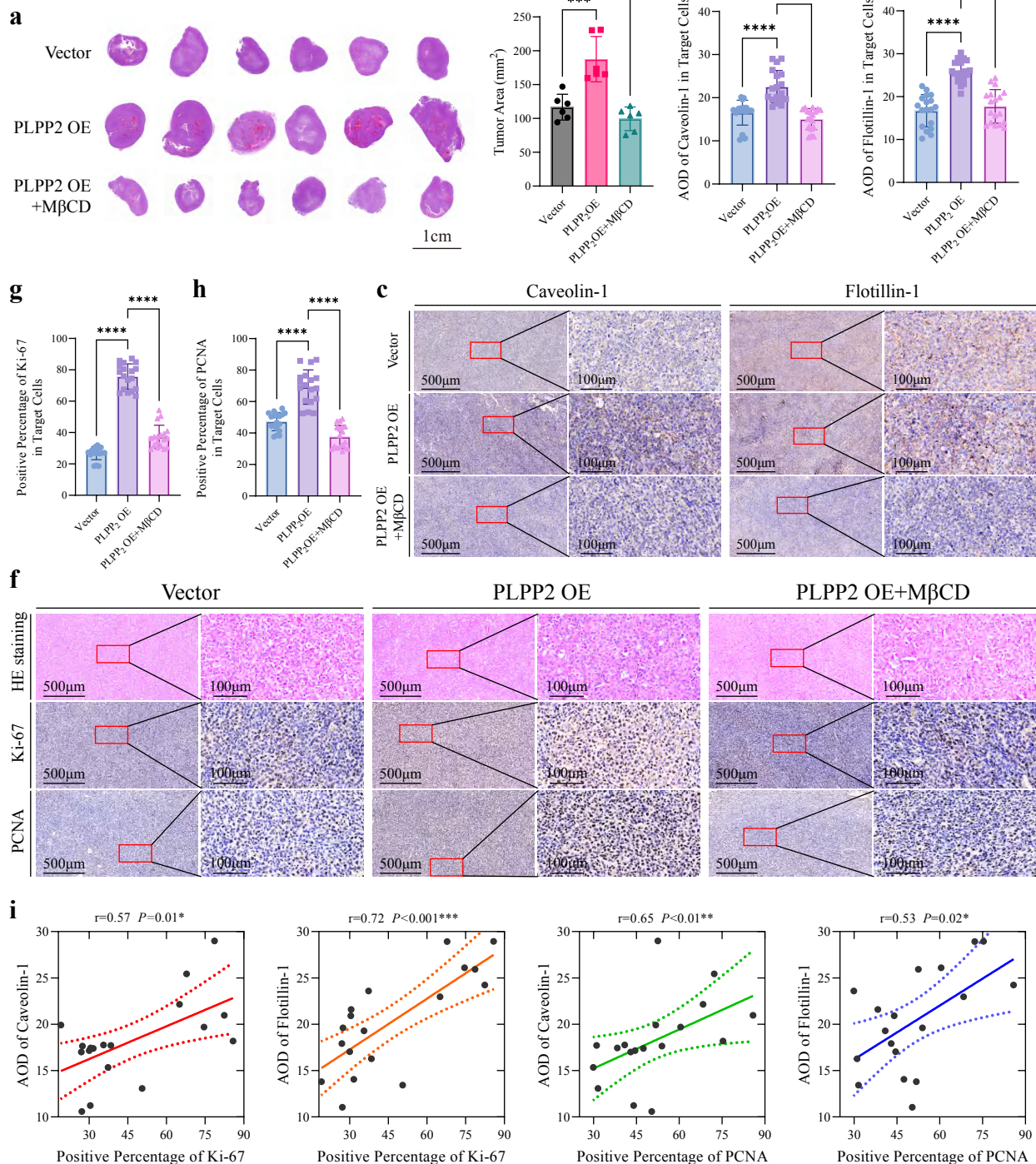

Supplement: Supplementary file 1 — Additional file 1. [file 13046_2023_2877_MOESM1_ESM.zip › Figure S8.pdf]
